# Emotional and behavioral problems, social competence and risk factors in 6–16-year-old students in Beijing, China

**DOI:** 10.1371/journal.pone.0223970

**Published:** 2019-10-24

**Authors:** Yang Yang, Yanjie Qi, Yonghua Cui, Bin Li, Zhixia Zhang, Yuming Zhou, Xu Chen, Dandi Zhu, Fan He, Yi Zheng

**Affiliations:** 1 The National Clinical Research Center for Mental Disorders & Beijing Key Laboratory of Mental Disorders, Beijing Anding Hospital, Capital Medical University, Beijing, China; 2 Advanced Innovation Center for Human Brain Protection, Capital Medical University, Beijing, China; 3 National Center for Children’s Health, Beijing, China; 4 Department of Psychiatry, Beijing Children’s Hospital, Capital Medical University, Beijing, China; International Telematic University Uninettuno, ITALY

## Abstract

**Introduction:**

Child emotional or behavioral problems and insufficient social development has been a heavy burden on family and society. However, currently large-scale studies on emotional and behavioral problems as well as social competence among school children in China are still lacking. This cross-sectional study analyzed the current status and risk factors of behavioral problems and social competences in Beijing students.

**Method:**

A total of 9,295 students, with ages ranging from 6 to 16 years old, were enrolled in the study. The Child Behavior Checklist (CBCL) was used to screen emotional and behavioral problems, social competences of students. We then assessed significant predictors factors associated with children behavioral problems and social competences.

**Results:**

The total detection rate of behavioral problems of this cohort was 16.7%. All kinds of social competence scores of boys were lower than girls (*P* <0.05). The scores of social and learning ability in children with behavioral problems were significantly lower than those without behavior problems (*P* <0.05). Gender, developmental delay, recent life events, negative relationships and negative child-rearing styles were the shared influencing factors for behavioral problems and social competence. In addition, age, macrosomia, threatened abortion, hospitalization for physical illness, physical illness, poor sleep were independent risk factors for children's emotional and behavioral problems, and non-breastfeeding was an independent risk factor for abnormal social competence.

**Conclusion:**

The social competence, emotional and behavioral problems are serious among students in Beijing. More attention should be paid to mental health and effective intervention measures should be provided.

## Introduction

In the contemporary society, school-age children and adolescents are confronted with a variety of psychological conditions which are frequently associated to negative consequences on the behavioral problems and social competence development. The behavioral problems refer to abnormal behaviors that exceed the normal range for the corresponding age in terms of severity and duration [1]. These behavioral problems arise during development and can lead to more serious behavioral deficits and emotional issues. Specifically, students aged 6–16 in school face the transition from childhood to early adolescence (from 12 to 14 years) [2] and numerous physical, emotional and cognitive changes are triggered by puberty occur. Adolescence begins with separation from parents, rebuilding of peer relationships [3], and confirmation of their personal, social, and sexual identities [4,5]. As children reach adolescence, they become increasingly self- reliant, engaging in planful problem solving and utilizing cognitive strategies more frequently when faced with stressful life events [6]. At this stage of development, the regulatory competencies, analyze emotion, emotion regulation, and executive function have substantial development [7,8]. However, the abilities functional to self-regulation are still relatively immature. These changes directly impact emotionality, affecting both the valence and intensity of negative and positive emotions. Although majority of the children and adolescents successfully navigate these developmental stages, for some, this becomes the beginning of emotion regulation and resultant dysregulation [7,9]. Children and adolescents with behavioral problems often have difficulties in expressing themselves and in understanding others’ emotions and motivation, which negatively impact their social and academic competence, and affect their career planning abilities [10–12]. Social competence, along with growth and cognitive competence in children are referred to effectiveness in social interactions. This is considered to be the overall ability of individuals to act in a socially appropriate manner [13]. Development of social competence has been regarded as the predictor of future behavioral and behavioral problems, as well as social adaptation [14]. Many studies suggest that optimal social competence can avoid unacceptable responses from others and reduce the inadaptability risk, disharmonious relationships, interpersonal conflicts [15,16]. Based on these findings, we believe it is necessary to pay special attention to the behavioral problems and social competence in school children and adolescents. Multiple studies regarding the prevalence of childhood behavioral problems and the mental health situation has been conducted. It is estimated that the global prevalence of mental disorders among children and adolescents is around 7–22%, with an incidence of 12.4–21.8% in developed countries and 10.4–37.6% in developing countries [17–19]. Several risk factors of behavioral problems have been studied in developed countries, which includes perinatal, social, family, and environmental factors. For example, moderately and late preterm birth leads to a greater risk of adverse childhood outcomes, including emotional and behavioral problems [20]. Some studies find the effects on neurodevelopment and behavioral disorders due to pre or postnatal exposure to arsenic, cadmium and manganese in children up to 16 years of age[21]. Sociological study shows that family income and parental education when entering preschool are significant predictors of mental health problems after elementary school enrollment [22]. Especially the latest research in psychiatric epigenetic has moved to more complex models of psychopathology incorporating a focus on gene–environment and epigenetics interactions. Several studies have underlined that children’s emotional and behavioral problems are predicted by children’s genetically-based features, and parental psychopathology is considered as one of the risk associated with epigenetic modifications in offspring [23,24]. Environmental risk factors have been shown to be crucial in affecting children’s mental health [25], and epigenetics foster the onset of both internalizing and externalizing problems through the interaction of the environment and genes [26,27]. Furthermore, these psychopathological symptoms in children has also been a life-long stability [28,29], which in turn would contribute to further intergenerational transmission of psychopathological risk. In China, some of the mentioned problems are prevalent and may influence the behavioral problems of Chinese children [30–34], but it is unclear whether they are predictive risk factors for the behavior of Chinese children. Furthermore, currently reliable studies on behavioral problems of Chinese school-age children and adolescents are still lacking. Particularly, in the last 5 years, there has been no large-scale study on behavioral problems and social competence among students in Beijing, which raises necessity for studies to illustrate. In order to explore this, our study tries to take a first step in direction, using the Child Behavior Checklist (CBCL) to screen behavioral problems and social competences of students in Beijing, and assessing the associated risk factors, to provide a scientific basis and preventive strategy for the implementation of relevant public health policies.

## Subjects and methods

### Study subjects

The current cross-sectional study is part of the national investigation of psychiatric disorder in China held by Beijing Anding Hospital. Beijing is divided into six urban districts and ten suburban districts according to economic and administrative planning. According to the 2013 Beijing Regional Statistical Yearbook, Xicheng as urban district (the gross domestic product (GDP) of 259.35 billion RMB, population of 1.385 million) and Huairou as suburban district (GDP of 15.32 billion RMB, population of 0.278 million) were selected for the part of this study. The two districts represent different socioeconomic statuses, regional development levels, and accurately demonstrate the differences between urban and suburban areas of Beijing. Inclusion criteria were 1) participants should be the resident of the selected study site; 2) children and adolescents enrolled in school ranging from 6 to 16 years old; and 3) children and their parents or guardians gave consent to participate. Exclusion criteria included 1) subjects who refused to sign the informed consent form; 2) students not attending school or in special schools, or 3) students who could not be contacted after at least three attempts to visit at different times.

The sample size of this study was based on the sample size of the national investigation of psychiatric disorder in China. The minimum sample size was calculated based on the prevalence of behavioral problems among children in China. According to the literature, the prevalence of any behavioral problems in children and adolescents is 10–30% [35]. Considering the prevalence 10% with error of 1% and 95% confidence interval, the minimum required sample size was 3458. Since we used nonrandom sampling method for selecting the study sites a design effect value of 2 was used for final sample size estimation with a probable non-response rate of 30%. The final required sample was about 9000. Multistage stratification and random sampling of the entire population were carried out to obtain samples. Schools were chosen at random, according to the number of primary schools and secondary schools in each district. Sixteen schools, including four urban secondary schools, eight urban primary schools, three rural secondary schools, and one rural primary schools, were selected at random. Classes from each Grades were randomly selected from each school, depending on the size of the school.[36,37] The participants were recruited between January 1, 2014 and December 31, 2014.

### Instruments

The CBCL parent-report is a validated questionnaire that assesses behavioral problems, emotional difficulties, and social competencies of a child [38]. The CBCL has test-retest reliability 0.95 for behavior problems, and shows good agreement between maternal and paternal rating [38–40] This standardized and objective measurement tool has been revised and widely used for clinical and research purposes [41,42]. The standardized Chinese version of CBCL/4-16 (1991, parental version) was used in our study to screen behavioral problems and social competence of students [41]. The CBCL/4-16 survey questionnaire is intended for children aged 4–16, including general questions as well as items related to social competence and behavioral problems. Social competence includes 3 parts: activity ability, social ability, and learning ability. The section on behavioral problems contains 113 questions divided into 9 factors, providing scores for three major scales: internalizing (sum of withdrawn, somatic complaints, and anxious/depressed subscales), externalizing (sum of attention problems and aggressive and delinquent behavior subscales), and total behavior problems. The sub-scales differ depending on age (4–5 years old, 6–11 years old and 12–16 years old, respectively) and gender. Each item is answered based on past 6 months experiences, and scored on a three-point Likert scale (0 = not true, 1 = somewhat or sometimes true, or 2 = very true or often true) [38]. The total score is the sum of various factor scores. The threshold value of the CBCL used in this study was based on the model for Chinese children and adolescents, and participants were considered to be screened positive when their score over the threshold value of each sub-scale or/and CBCL total scale S1–S4 Tables. The scale has been well standardized, and various studies have found it to have good reliability and validity. The test-retest reliability and criterion validity of Chinese version CBCL for total behavior problems is 0.83, 0.85, respectively [41]. In the current study, Cronbach alpha value coefficient for total behavior problems was 0.97.

### Sociodemographic characteristics

A general questionnaire concerning psychological factors of children was designed by the authors specifically for this study. Primary caregivers of the children were asked at least 49 questions in order to provide information on: (1) sociodemographic features of the parents and the family (including region, gender, age, ethnicity, religion, sibling situation); (2) growth and developmental features, medical and psychiatric history of the child (including perinatal status, maternal pregnancy health, pregnancy and birth status, birth pattern, birth time, birth weight, neonatal health status, feeding methods, growth and development, physical illness, sleep); (3) general status of the family and possible social stress factors (including family structure, parenting child rearing style, primary caregiver, kinship, family relationship, family physical and mental health history, family history of suicide, parental occupation, life events).

### Study process

This study was approved by the institutional ethics review board of Beijing Anding Hospital of Capital Medical University. The researchers included physicians and graduate students from the psychiatry department as well as staff who were responsible for quality control. Firstly, training and a pilot experiment was done. The training covered various aspects such as: background of the project, relevant knowledge, principles and methods of sampling design, questionnaire administration, and on-site investigation methods and processes. The investigators explained the questionnaire and answering methods to parents (including father or mother, or primary caregiver of the child) during class meetings. The questionnaire for each child was only administered to one single guardian. In the content of the inquiry, each question must have a clear answer and cannot be ambiguous. Questionnaire distributed and collected by investigators, and centrally maintained to ensure confidentiality. Staff members were responsible for entering raw data using double entry, and 10% of the questionnaires were selected for review.

### Statistical analysis

Epidata 3.1 was used to create the database. All data were analyzed using SPSS 22.0, (IBM Inc. Armonk, NY, 2013). Basic statistical description of the survey information was conducted with normally distributed quantitative data analyzed using *t*-tests; categorical data were analyzed using *χ*^*2*^ tests. For non-normal distribution, non-parametric test was used. In this study, univariate analysis and multivariate logistic regression were used to identify the influencing factors of behavioral problems and social competence. Univariate analysis was performed by *t*-tests and χ^2^ tests. Based on the significant associations in univariate analysis, multivariate logistic regression analysis was performed to identify the associated risk factors adjusting for age, sex and other covariates.

## Results

A total number of 10327 students was enrolled, of which 9478 participants (91.8%) gave consent to participate. Of the eligible participants, we obtained valid data from 9295 students, with a response rate of 98.1%.

### Participant characteristics

The information was available for 9295 students, with age ranging from 6 to 16 years (mean: 11.04±2.84 years). There were 4802 boys (51.7%) and 4493 girls (48.3%). According to the division of early childhood and adolescence age (from 12 years) and CBCL age group, all students are divided into two different age groups: 6–11 years and 12–16 years The differences in gender distribution among different age groups were not statistically significant (*χ²* = 16.8; p = 0.08).

A total of 51.1% of the students lived in nuclear families and 54% of the students came from suburban district. Most stayed with their parents (95.6%) and 71% had no siblings. The fathers of 9.6% of the students were unemployed while the mothers of 25.9% were unemployed. Most child-rearing styles were of democratic (40.7%) or mixed types (51.3%), with 1.1% using violence child-rearing style. However, 7.7% of the information for this section was missing. The complete-case analysis was used.

### Detection rate of behavioral problems

The total detection rate of behavioral problems from the two districts in Beijing (Xicheng and Huairou) was 16.7%. The prevalence of behavioral problems in boys and girls were 13.2% and 20.3% respectively and the difference was statistically significant (χ² = 84.17, p<0.001) (Table 1). In stratified analysis according to age and gender, we found that girls had a significantly higher detection rate of total behavioral problems than boys of in 6–11 age group (*χ*^*2*^ = 142.98, p<0.001). Nearly 4.4% of boys had social problems and nearly 26% girls had depression in 6–11 age group. No significant difference in total behavioral problems were seen across the gender among 12–16 age students (*χ²* = 1.13; p = 0.288). (Table 2).

**Table 1:** Differences in sociodemographic characteristics for detection rate of behavioral problems and total CBCL scores in students.

Variable	Groups	Total(N)	Behavioral problems		Total CBCL score	Z/H
			n (%)	$x^{2}$	(Median, IQR)	
**Gender**	Boys	4802	636 (13.2)	84.17**	10.00, 22.00	-5.45**
	Girls	4493	914 (20.3)		9.00, 19.00	
**Age**	6-11years	4329	827 (19.1)	33.73**	10.00, 22.00	-0.59
	12–16 years	4966	725 (14.6)		9.00, 22.00	
**District**	Suburban	5012	851 (17.0)	0.82	10.00, 21.00	-4.31**
	Urban	4270	695 (16.3)		9.00, 19.00	
**Parturition timing**	Termneonate	8172	1317 (16.1)	18.43**	9.00, 20.00	42.85**
	Premature birth	542	105 (19.4)		13.00, 23.00	
	Post-term delivery	534	121 (22.7)		13.00, 23.00	
**Birth weight**	Normal	8685	1423 (16.4)	6.37*	10.00, 21.00	16.35**
	Low	332	63 (19.0)		11.00, 22.00	
	Macrosomia	268	58 (21.6)		13.00, 26.00	
**Threatened abortion**	No	8759	1431 (16.3)	17.19**	9.00, 21.00	-5.98 **
	Yes	469	111 (23.7)		13.00, 22.00	
**Feeding method**	Breastfeeding	5760	924 (10.8)	4.23*	9.00, 20.00	-4.49**
	Non-breastfeeding	3506	620 (17.7)		11.00, 21.00	
**Developmental delay**	No	8896	1425 (16.0)	94.18**	9.00, 20.00	-10.73**
	Yes	297	111 (37.4)		25.00, 35.00	
**Relationship with family**	Harmonious	8204	1210 (14.7)	205.40**	9.00, 19.00	-18.05**
	Disharmonious	1046	338 (32.3)		22.00, 33.00	
**Relationship with surrounding**	Harmonious	7774	1109 (14.3)	210.88**	8.00, 18.00	-18.01**
	Disharmonious	1469	436 (29.7)		19.00, 30.00	
**Sleep**	Good	8965	1442 (16.1)	90.03**	9.00,20.00	-10.69**
	Bad	261	100 (38.3)		26.00, 36.00	
**Hospitalization for physical illness**	No	1989	1259 (15.8)	42.72**	9.00, 20.00	-9.05**
	Yes	1226	285 (23.2)		13.00, 26.00	
**Physical illness**	No	7744	1188 (15.3)	66.24**	9.00, 20.00	-10.92**
	Yes	1487	356 (23.9)		14.00, 25.00	
**Recent life events**	No	8048	1133 (14.1)	309.67**	8.00, 18.00	-22.13**
	Yes	1159	402 (34.7)		23.00, 33.00	
**Child-rearing styles**	Democratic	3731	434 (11.6)	130.60**	6.00, 15.00	340.94**
	Violent	104	25 (24.0)		15.00, 23.00	
	Pampering	549	114 (20.8)		12.00, 27.00	
	Conflicting	91	31(34.1)		23.00,35.00	
	Mixed	4720	932(19.7)		12.00,23.00	

*P value was significant at <0.05,

**P value was significant at <0.01. Z-test or H-test. IQR- Inter Quartile Range.

**Table 2:** Detection rate of behavioral problems in students.

6–11 years old			12–16 years old	
Variable	Boys *n* = 2297 (%)	Girls *n* = 2032 (%)	Boys *n* = 2505 (%)	Girls *n* = 2461 (%)
**Aggressive behavior**	40 (1.7)	19 (0.9)	97 (3.9)	117 (4.8)
**Rule-breaking behavior**	24 (1.0)	14 (0.7)	74 (3.0)	96 (3.9)
**Somatic complaints**	24 (1.0)	15 (0.7)	80 (3.2)	81 (3.3)
**Hyperactivity**	43 (1.9)	38 (1.9)	140 (5.6)	NA
**Depression**	40 (1.7)	525 (25.8)	NA	NA
**Social withdrawal**	42 (1.8)	39 (1.9)	NA	NA
**Schizoid disorders**	71 (3.1)	NA	87 (3.5)	218 (8.9)
**Social problems**	101 (4.4)	NA	98 (3.9)	NA
**Obsessive- compulsive**	65 (2.8)	NA	118 (4.7)	NA
**Cruelty**	NA	20 (1.0)	102 (4.1)	NA
**Sexual problems**	NA	55 (2.7)	NA	NA
**Schizoid-compulsive**	NA	44 (2.2)	NA	NA
**Anxiety-Compulsive**	NA	NA	NA	93 (3.8)
**Hostility**	NA	NA	82 (3.3)	NA
**Immaturity**	NA	NA	82 (3.3)	102 (4.1)
**Depression/ withdrawal**	NA	NA	NA	109 (4.4)
Total behavioral problems*#	284 (12.4)*	542 (26.7)*	352 (14.1)#	372 (15.1)#

*Chi square test value 142.98 (P value < 0.001).

#Chi square test value 1.13 (P value = 0.288).

NA- Not Applicable.

### CBCL scores for students

The various CBCL factor scores for students according to age and gender is shown in Table 3. In the 6–11 age group, the aggression factor scores were higher than other factors among both boys and girls. Apart from aggression, the factor scores for hyperactivity problems were high for boys while the factor scores for depression were high for girls. The total CBCL score was significantly higher for boys than girls (*Z* = -5.89; p*<*0.001), which indicates that the boy’s behavior problem is more serious. In the 12–16 age group, among these factors, aggression, hyperactivity, and social problems had the highest scores for boys, while aggression and anxiety-compulsive factors were the factors with the two highest scores for girls.

**Table 3:** Behavioral problem factor scores for students.

6–11 years old			12–16 years old	
Variable	Boys (*n* = 2297) (Median, IQR)	Girls (*n* = 2032) (Median, IQR)	Boys (*n* = 2505) (Median, IQR)	Girls (*n* = 2461) (Median, IQR)
**Aggressive behavior**	3.00, 6.00	2.00, 5.00	2.00, 5.00	2.00, 6.00
**Rule-breaking behavior**	0.00, 2.00	0.00, 0.00	0.00, 2.00	1.00, 4.00
**Somatic complaints**	0.00, 1.00	0.00, 2.00	0.00, 2.00	0.00, 1.00
**Hyperactivity**	2.00, 4.00	1.00, 3.00	2.00, 4.00	NA
**Depression**	0.00, 2.00	2.00, 4.00	NA	NA
**Social withdrawal**	0.00, 1.00	1.00, 3.00	NA	NA
**Schizoid disorders**	1.00, 2.00	NA	0.00, 2.00	0.00, 1.00
**Social problems**	1.00, 2.00	NA	1.00, 2.00	NA
**Obsessive- compulsive**	1.00, 3.00	NA	1.00, 2.00	NA
**Cruelty**	NA	0.00, 0.00	NA	0.00, 0.00
**Sexual problems**	NA	1.00, 2.00	NA	NA
**Schizoid- compulsive**	NA	0.00, 1.00	NA	NA
**Hostility**	NA	NA	1.00, 3.00	NA
**Immaturity**	NA	NA	0.00, 2.00	1.00, 4.00
**Depressed/ withdrawn**	NA	NA	NA	1.00, 4.00
**Anxiety- compulsive**	NA	NA	NA	1.00, 4.00
Total score (Range)*#	11.00, 22.00*	9.00, 17.00*	10.00, 22.00#	9.00, 21.00#

*Z-test for 6–11 years (-5.89, p<0.001).

#Z-test for 12–16 years (-2.13, p = 0.033).

NA- Not Applicable, IQR- Inter Quartile Rang.

### Differences in sociodemographic characteristics for detection rate of behavioral problems and total CBCL scores

Table 1 represents the behavioral problems across various socio-demographic factors. Significant association was seen with gender, age groups, parturition timing, birth weight, feeding method, development, relationship, sleep, life events, physical illness and child-rearing styles (p<0.05).

The differences in sociodemographic characteristics for total CBCL scores in students is shown in Table 1. The distribution of sociodemographic characteristics for CBCL scores was generally consistent with the distribution of detection rates.

### Social competence abnormalities

**Fig 1:**
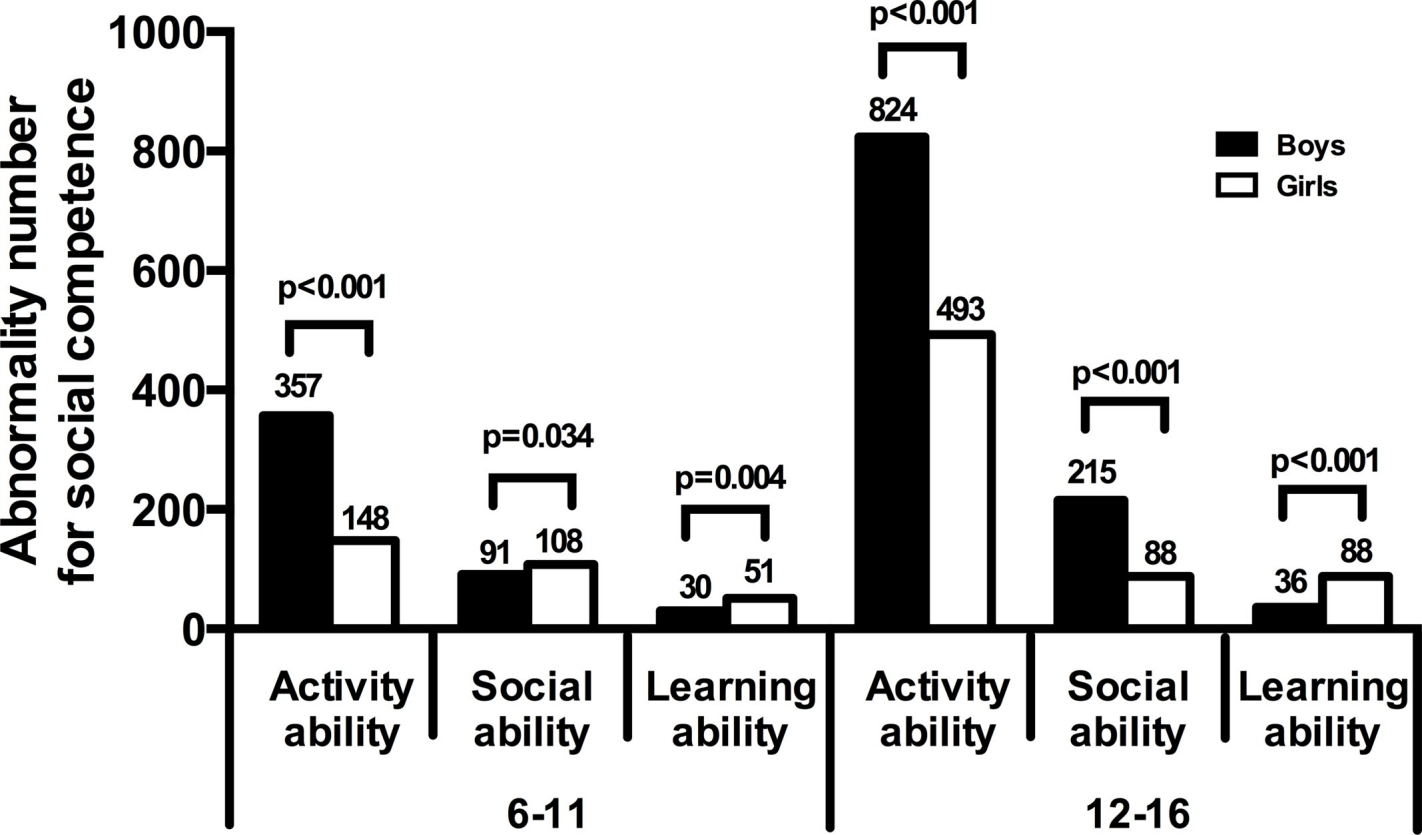
Comparison of abnormal numbers for social competence in students.

**Fig 2:**
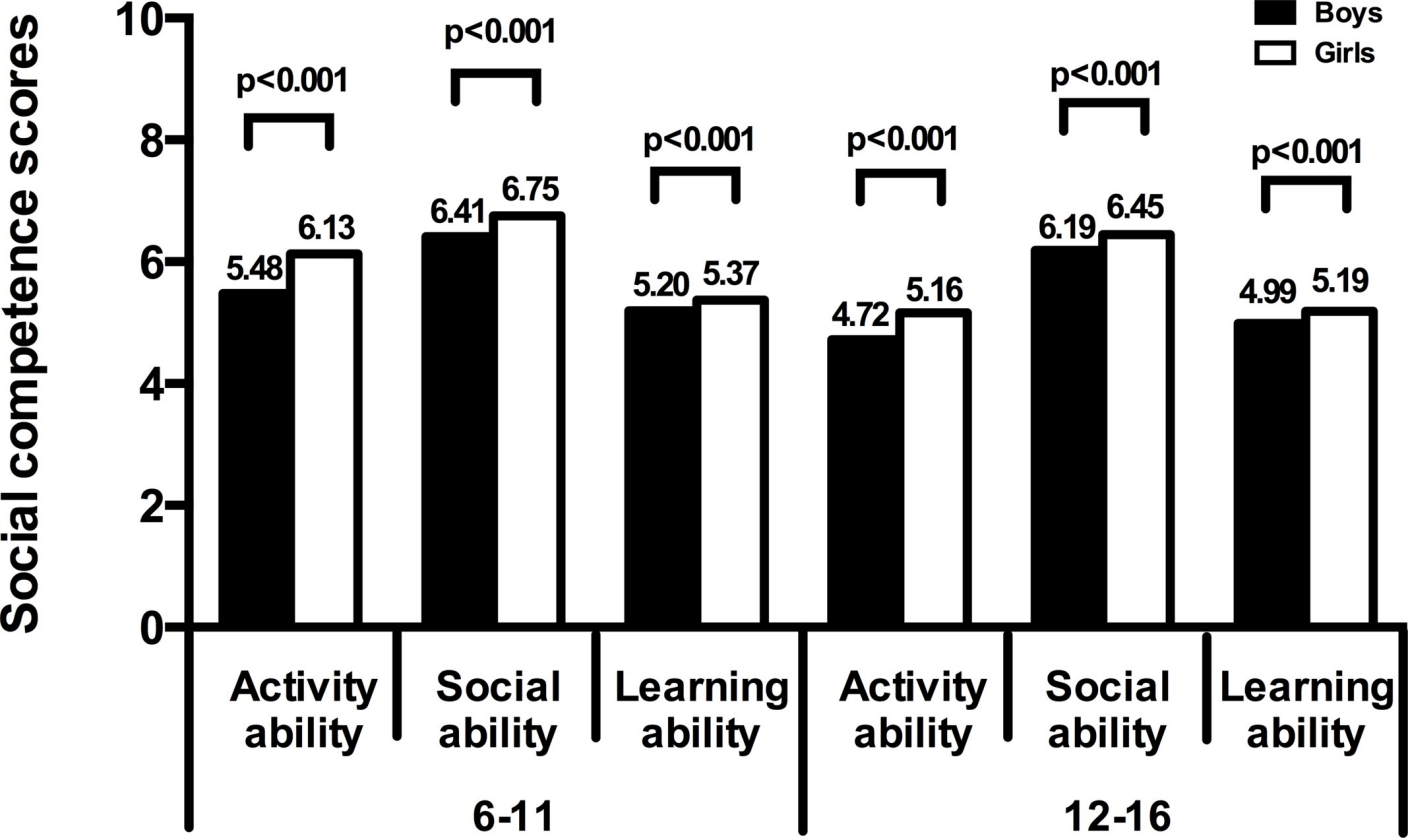
Comparison of social competence scores in students.

In different age groups, boys had higher abnormal activity than girls, while girls had more abnormal learning ability than boys (Fig 1). Boys from different age groups had obviously lower activity ability, social ability, and learning ability scores than girls (Fig 2). The scores of social ability (6.19±2.08), and learning ability (4.97±0.87) in children with behavioral problems were all lower than those of children without behavioral problems (the scores were 6.48±2.10; 5.22±0.80, respectively); these differences were statistically significant (t = 5.05, 0.65, respectively; p<0.01).The scores of activity ability were no statistically different between children with behavioral abnormalities and without behavioral abnormalities.

### Risk factors of behavioral problems and social competence

Multivariate logistic regression analysis was performed on the statistically significant variables (p<0.05) in the single factor analysis results. Analysis results of the behavioral problems showed that in the demographic characteristics, age was a possible protective factor for behavioral problems in children and adolescents and female was a risk factor. On the growth and development level, threatened abortion, macrosomia, developmental delay, hospitalization for physical illness, physical illness in the past year, and poor sleep may be risk factors for behavioral problems in children and adolescents. On the family level, poor interpersonal relationships with family and surrounding people, pampering, contradictory and mixed child-rearing style, and recent life events were risk factors for behavioral problems (Table 4). The Table 4 showed the influencing factors affecting children's social abilities, especially non-breastfeeding was the common risk factor for children's social abilities. At the same time, gender, developmental delay, recent life events, negative relationships and negative child-rearing styles were the shared influencing factors for behavioral problems and social competence.

**Table 4:** Logistic regression analysis of risk factors affecting behavioral problems and social competence.

Risk factor		Behavioral problems *OR* [95% *CI*]	Social competence		
			Activity ability	Social ability	Learning ability
			*OR* [95% *CI*]	*OR* [95% *CI*]	*OR* [95% *CI*]
**Female**		2.08 [1.84–2.35]**	0.50 [0.44–0.56]**	0.76 [0.62–0.93]**	2.95 [2.09–4.14]**
**Developmental delay**		2.23 [1.70–2.93]**	1.73 [1.32–2.28]**	2.64 [1.85–3.77]**	3.69 [2.25–6.05]**
**Recent life events**		2.65 [2.27–3.10]**	1.27 [1.08–1.49]**	1.49 [1.16–1.91]**	1.90 [1.30–2.78]**
**Disharmoni-ous relationship**	with family	1.72 [1.43–2.06]**	1.23 [1.038–1.46]*		
	with surrounding	1.75 [1.49–2.05]**	1.81 [1.56–2.11]**	2.28 [1.83–2.84]**	2.44 [1.73–3.44]**
**Negative child-rearing styles**	Violent			4.15 [2.22–7.75]**	
	Pampering	1.62 [1.26–2.09]**	1.60 [1.27–2.03]**	2.26 [1.51–3.37]**	1.97 [1.04–3.74]*
	Conflicting	2.07 [1.25–3.44]**		2.53 [1.22–5.26]*	
	Mixed	1.58 [1.38–1.80]**	1.37 [1.21–1.55]**	1.84 [1.45–2.34]**	1.87 [1.289–2.74]**
**Threatened abortion**		1.37 [1.07–1.74]*			
**Macrosomia**		1.61 [1.16–2.22]**			
**Poor sleep**		2.3 [1.73–3.151]**			
**Physical illness**		1.43 [1.22–1.66]**			
**Hospitalization for physical illness**		1.31 [1.11–1.56]**			1.75 [1.19–2.58]**
**Age**		0.93 [0.91–0.95]**	1.10 [1.06–1.14]**	1.07 [1.03–1.12]**	
**Paternal employment**			0.80 [0.67–0.96]*	0.60 [0.45–0.80]**	
**Non-breastfeeding**			1.19 [1.06–1.34]**	1.62 [1.32–1.97]**	1.95 [1.42–2.67]**
**Urban district**			0.56 [0.46–0.69]**	1.74 [1.40–2.15]**	1.98 [1.44–2.73]**
**Maternal employment**			0.78 [0.68–0.89]**		
**Post-term delivery**			1.34 [1.06–1.68]*		
**Family history of mental illness**			3.17 [1.33–7.54]**		
**Living with parents**			1.63 [1.26–2.10]**		
**Not only child**				0.66 [0.52–0.85]**	

*P value was significant at <0.05,

**P value was significant at <0.01.

## Discussion

### The effects of age and gender on prevalence

Many studies have shown that there are age and gender differences in the severity and frequency of children’s behavioral and emotional problem. However, the impact of age and gender differences are rather inconsistent [43,44]. Other studies suggest that the internalizing and externalizing problems are co-occurrence in different genders children, and there is no difference between boys and girls with respect to externalizing and internalizing problems [2,45]. Our study found that the total detection rate for behavioral problems among 6-16-year-old students in two districts in Beijing was 16.7%. With age increasing, the incidence of behavioral problems showed decreasing trends. Boys mostly had poor social problems, with aggression and hyperactivity being more serious, while among girls, depression and anxiety had higher incidence and severity. This is in line with the results of previous studies, which have shown that the factors in boys are mainly externalizing behavioral factors, such as aggressive behavior, social problems, hyperactivity, and rule-breaking behavior, while girls have more internalizing factors, such as depression and withdrawal, anxiety [46,47]. One of the major findings of our present study is that the differences in behavioral problems among boys and girls were more significant in the 6–11 age group. Specifically, in the 6–11 age group, girls had more frequent depression and total behavior problems. However, the differences became insignificant in the 12–16 age group.

There are several reasons contributing to these phenomena. Firstly, children of different genders may have differences in cognitive, emotional, personality and social development [48,49]. Secondly, the majority of onset distributions of commonly occurring children mental disorders (including attention-deficit/ hyperactivity disorder (ADHD), oppositional-defiant disorder (ODD), and impulse- control disorders) begins in childhood, which makes behavior problems more prominent in 6–11 age group [50–52]. Finally, some study found that depression occurs in childhood without gender differences, but with puberty arriving, the prevalence of women is usually higher than that of men [53,54]. Previous researches propose the theoretical mechanism model that the biological factors (including genetic vulnerability, pubertal hormones, pubertal timing, development, and hypothalamic-pituitary-adrenal axis regulation) are vulnerabilities to depression that heighten girls' rates of depression beginning in adolescence and account for the gender difference in depression [55–58]. Girls undergo puberty earlier than boys, and these biological changes may affect the individual's sensitivity to stress. Females are more vulnerable to developing depression and related anxiety disorders than males [43,59–61]. Specifically, more research show that the pubertal timing advanced to before eight years of age in girls, and the total detection rate of precocious puberty in girls is significantly higher than that of boys. Young girls who had been early growth had higher rates of behavior problems compared with girls who were on-time growth, such as depression, anxiety, attacks, illegal behavior, substance abuse, eating disorders, premature sexual behavior and so on [62]. This trend also coincides with the different emotional behavior characteristics in developmental stage of adolescence and childhood. Adolescence is a period characterized by marked changes occurring on a hormonal, neurological, and developmental level [7]. During adolescence numerous changes occur in emotion and cognition. The emotion-activating experiences in development of adolescents’ brain could interfere with the regulation of emotions and in the evaluation of the risk, and could be responsible for adolescents’ general tendency to risk taking and impulsivity [2, 63–66]. Early adolescence may be considered a critical phase, during which emotional behavioral problems tend to become chronic disorders and forecasts many subsequent psychosocial impairments [3,67]. Results show that adolescents had higher levels of depression, obsession-compulsivity, paranoid ideation, and hostility than children[2]. In addition, influenced by the traditional Chinese concept of family preference, parents give different demands and concern to children with different genders in the family. This may influence children’s psychological development and lead to different behavioral problems in children with different genders. However, with the progression of school, the school becomes the main social environment, and the influence of family on children gradually weakens. Therefore, the differences in behavioral problems gradually become not obvious when children reach the secondary school.

In our study boys' social competence scored lower than girls, which suggested that boy’s social competence development worse than girls. There could be a number of reasons for this. First, our results showed that boys have more externalizing behavioral problems than girls—especially hyperactivity, aggressive behavior, and rule-breaking behavior—and these problems will manifest as attention deficit, impulsiveness, aggression, or a lack of self-control. These behavioral problems may be one of the reasons why boys score lower than girls in social competence and may affect the development of boy’s social competence. Second, boys and girls show differences in psychological development. Especially in childhood, boys are disadvantage at the psychological cognitive development in children such as mathematical ability, verbal skills, and spatial ability [68–71].

Boys are significantly more likely to have problems with adaptive behavior and social competence [72]. Generally, it’s been considered that mental capability develops along with aging in children. However, we found that social competence in children did not increase with age. Rather, the detection rates of social competence abnormalities showed increasing trends and scores of social competence showed slight decline with age. These may be due to the fact that in the current social environment of China, most children do not have siblings. Outside of school, most children have fewer opportunities to interact with peers, which may cause them to have a lack of opportunities to practice social abilities and activities [73]. At the same time, although children's mental illness begins in early childhood, the family environment is relatively loose or the abnormal performance has not been paid attention. After entering school, various restrictions have gradually emerged, especially with the pressure of education and social relations, the social competence abnormalities gradually appeared.

Overall, we used CBCL to assess the distribution of emotional and behavioral problems among children and adolescents. However, our current study uses only parental ratings, and didn’t use the Youth's Self-Report (YSR) and Teacher's Report Form (TRF) to evaluate children's emotional and behavioral problem. There is no information directly from students and teachers. Some researchers believe there have the high degree of agreement among parents, teachers and adolescents with report to the CBCL, TRF, and YSR. The original versions in English have good test-retest reliability and internal consistency and have strong criterion-related validity [38,74]. However, other clinical studies have found that correlations between child and parent or teacher reports to be small or medium at best [75–77]. Based on parent reports, children are found to have higher rates of internalizing problems than externalizing problems, which is the opposite of the teacher's report. This suggests that parents are more aware of emotional changes in their children, whereas teachers may be more likely to detect behavioral problems [78,79]. Parents and teachers see children in different situations and have different emotional relationships and expectations of the child. Hence, our results might overestimate the actual prevalence of internalizing problems, such as anxiety and depression. So it is not comprehensive to assess the behavioral problems of parents alone. Using CBCL in conjunction with YSR and TRF to assess the behavioral problems from different perspectives of reporters is more conducive to the timely and comprehensive detection of behavioral problems.

### Risk factors

We conducted risk factor analysis for growth and development status and found that using medication during pregnancy, developmental delay; physical illness and poor sleep are among risk factors, most of which are consistent with previous reports. Interestingly, our results considering birth weight are different. Many previous studies have shown that low birth weight is a causal risk factor for child problem behavior, the effects of which may well extend into adulthood [80–82]. Our study, however, found that macrosomia could be a risk factor for emotional and behavioral problems in children. Macrosomia associates with the externalizing problems and the proportion of slow temperament type is greater among children with fetal macrosomia than among those with normal birth weight. This temperament characteristic predisposes children to many types of behavioral problems. Children with fetal macrosomia have poorer satisfaction in the cognitive component than children with normal birth weight. This affects their quality of life, and they have more severe sensory integration dysfunction than children with normal birth weight. Overweight children often manifest low self-esteem, depression, anxiety, and decreased social competence. These psychosocial problems contribute to the overweight child’s maladjustment to the demands of increasing social interactions within the family and the environment. Such evidence helps to illustrate that macrosomia as a risk factor for Emotional and behavioral problems [83–86]. To the best of our knowledge, this is the one study to directly suggest that macrosomia being a risk factor for childhood Emotional and behavioral problems, which is of greater importance nowadays, considering that with the increasingly improved living standards in recent years, the average birth weight of newborns has begun to increase [87] and the prevalence of fetal macrosomia has increased significantly [88].The higher rate of behavioral problems and psychopathology among macrosomia indicated overweight prevention and treatment are also critical to mental health in adolescents. Therefore, our study of risk factors may help to the development of targeted intervention and prevention programs.

More importantly, we found that feeding methods affect overall social competence since breastfed children have better development of activity, social ability, and learning ability. A study by Bier et al. found that the cognitive and motor scores of premature babies who were breastfed were higher than those of babies fed with formula [89]. In addition, Morley et al. found that at 18 months, breastfed babies had better mental and psychomotor development than babies who received formula [90]. The breastfeeding process is one of intimate contact between mother and child. Intimate mother-and-child contact during breastfeeding may have important effects on the infant’s cognitive development that are favorable for producing a sense of security in the world. This is also one reason that feeding method may be a risk factor affecting child behavioral problems.

It is worth mentioning that a growing amount of research has underlined the complex interplay between genes and the environment in shaping children’s development and emotional/behavioral adaptive functioning [91–93]. Recent studies have provided evidence that epigenetic is a possible mechanism by which environment interacts with genes and produces behavioral changes. It has been proposed that epigenetic alterations are biological responses to environmental factors. The effect of gene polymorphisms and promoter methylation associated with psychopathological symptoms outcomes, vary depending on environmental factors [23,94]. These study supports the emergent idea that exposure during childhood to potentially negative experiences (such as marital conflict, parental psychopathological risk, and poor quality of parent–infant relationships [95–97]) acts as an environmental risk factor for triggering children’s DNA methylation, and further fostering the onset of emotional and behavioral problems in children [98,99]. And these negative experiences during childhood, which may be risk factors associated with epigenetic modifications, are partially in line with the environmental and social risk factors of the children’s emotional-behavioral problems in our study. Then our research attempts to identify the intervening risk factors and provide scientific evidence for preventive strategies for emotional and behavioral problems in children.

Overall, there are substantial behavioral and emotional problems in 6-16-year-old students in Beijing. These problems are as common as other studies that used the CBCL and in high-income countries. Based on this survey we found risk factors which can be intervened to prevent behavioral and emotional problems in children. These should be the key risk factors which should be the main focus for interventions. It is necessary to establish future research to identify psychopathological risks in later life and reducing the negative impact of behavioral and emotional problems by controlling risk factors.

### Limitations

First, we did not compare differences between normal schools and key schools, as the inconsistencies between them are difficult to identify. Second, only students enrolled in mainstream schools were included, and children in special schools or who did not go to school were not included. Thus, the data was extracted from the large study of national and the findings cannot well represent all children and adolescents. Furthermore, CBCL is a self-assessed screening scale for evaluation of emotional-behavioral problems and social problems. Future studies should use semi-structured or structured interviews to obtain diagnosis and prevalence of psychosis. In additional, we think that a great limitation of the study derives from having information only from caregivers and there is no information directed to students or teachers. Therefore, it is recommended to use CBCL in conjunction with YSR and TRF to more fully reflect the behavior of young people.

## Supporting information

S1 Table Threshold value of each sub-scale and CBCL total scale in 6–11 years old boy behavior problem. (DOC)

S2 Table Threshold value of each sub-scale and CBCL total scale in 6–11 years old girl behavior problem. (DOC)

S3 Table Threshold value of each sub-scale and CBCL total scale in 12–16 years old boy behavior problem. (DOC)

S4 Table Threshold value of each sub-scale and CBCL total scale in 12–16 years old girl behavior problem. (DOC)
