# Supplementary material for: Emotional and behavioral problems, social competence and risk factors in 6–16-year-old students in Beijing, China
Source: PLoS One. 2019 Oct 24;14(10):e0223970. doi: 10.1371/journal.pone.0223970 (PMC6812843; doi:10.1371/journal.pone.0223970)
Supplement: S1 Table — (DOC) [file pone.0223970.s001.doc]

**S1 Table**

**Threshold value of each sub-scale and CBCL total scale in 6-11 years old boy behavior problem （According to the data obtained from pre-experimental and norm synthesis）**

| **Factors** | **Schizoid disorders** | **Depression** | **Social problems** | **Obsessive- compulsive** | **Somatic complaints** | **Social withdrawal** | **Hyperactivity** | **Aggressive behavior** | **Rule-breaking behavior** | **Total behavioral problems** |
| --- | --- | --- | --- | --- | --- | --- | --- | --- | --- | --- |
| **Threshold value** | 5 | 9 | 5 | 8 | 6 | 5 | 10 | 19 | 7 | 40 |
