# Supplementary material for: Emotional and behavioral problems, social competence and risk factors in 6–16-year-old students in Beijing, China
Source: PLoS One. 2019 Oct 24;14(10):e0223970. doi: 10.1371/journal.pone.0223970 (PMC6812843; doi:10.1371/journal.pone.0223970)
Supplement: S2 Table — (DOC) [file pone.0223970.s002.doc]

**S2 Table**

**Threshold value of each sub-scale and CBCL total scale in 6-11 years old girl behavior problem （According to the data obtained from pre-experimental and norm synthesis）**

| **Factors** | **Depression** | **Social withdrawal** | **Somatic complaints** | **Schizoid-**  **compulsive** | **Hyperactivity** | **Sexual problems** | **Rule-breaking behavior** | **Aggressive behavior** | **Cruelty** | **Total behavioral problems** |
| --- | --- | --- | --- | --- | --- | --- | --- | --- | --- | --- |
| **Threshold value** | 3 | 8 | 8 | 3 | 10 | 3 | 2 | 18 | 3 | 37 |
