# Supplementary material for: Emotional and behavioral problems, social competence and risk factors in 6–16-year-old students in Beijing, China
Source: PLoS One. 2019 Oct 24;14(10):e0223970. doi: 10.1371/journal.pone.0223970 (PMC6812843; doi:10.1371/journal.pone.0223970)
Supplement: S3 Table — (DOC) [file pone.0223970.s003.doc]

**S3 Table**

**Threshold value of each sub-scale and CBCL total scale in 12-16 years old boy behavior problem （According to the data obtained from pre-experimental and norm synthesis）**

| **Factors** | **Somatic complaints** | **Schizoid disorders** | **Social problems** | | **Immaturity** | **Obsessive- compulsive** | **Hostility** | **Rule-breaking behavior** | **Aggressive behavior** | **Hyperactivity** | **Total behavioral problems** |
| --- | --- | --- | --- | --- | --- | --- | --- | --- | --- | --- | --- |
| **Threshold value** | 10 | 7 | | 14 | 5 | 5 | 10 | 8 | 18 | 9 | 38 |
