# Supplementary material for: Emotional and behavioral problems, social competence and risk factors in 6–16-year-old students in Beijing, China
Source: PLoS One. 2019 Oct 24;14(10):e0223970. doi: 10.1371/journal.pone.0223970 (PMC6812843; doi:10.1371/journal.pone.0223970)
Supplement: S4 Table — (DOC) [file pone.0223970.s004.doc]

**S4 Table**

**Threshold value of each sub-scale and CBCL total scale in 12-16 years old girl behavior problem （According to the data obtained from pre-experimental and norm synthesis）**

| **Factors** | **Anxiety-**  **Compulsive** | **Somatic complaints** | **Schizoid disorders** | **Depression/ withdrawal** | **Immaturity** | **Rule-breaking behavior** | **Aggressive behavior** | **Cruelty** | **Total behavioral problems** |
| --- | --- | --- | --- | --- | --- | --- | --- | --- | --- |
| **Threshold value** | 17 | 7 | 3 | 12 | 11 | 11 | 7 | 4 | 37 |
